# Intrinsic and Extrinsic Factors Impacting Cancer Stemness and Tumor Progression

**DOI:** 10.3390/cancers14040970

**Published:** 2022-02-15

**Authors:** Alexey Ponomarev, Zarema Gilazieva, Valeriya Solovyeva, Cinzia Allegrucci, Albert Rizvanov

**Affiliations:** 1 Institute of Fundamental Medicine and Biology, Kazan Federal University, 420008 Kazan, Russia; alesponomarev@stud.kpfu.ru (A.P.); zaregilazieva@kpfu.ru (Z.G.); vavsoloveva@kpfu.ru (V.S.); 2 School of Veterinary Medicine and Science (SVMS) and Biodiscovery Institute, University of Nottingham, Nottingham NG7 2RD, UK; cinzia.allegrucci@nottingham.ac.uk

**Keywords:** oncological diseases, cancer stem cells, stemness, spheroids, drug screening, tumor microenvironment

## Abstract

**Simple Summary:**

Presently, the study of cancer stem cells is important because these cells increase the cancer complexity, confer tumors the ability to grow, resist treatment, and survive in adverse conditions. One of the properties that these cells have is stemness. Cancer stemness is modulated by the tumor microenvironment, which influences cancer stem cell function and survival. This review includes information about cancer stem cells and their regulation by extrinsic and intrinsic factors. Pluripotency factors and signaling pathways, which regulate and modulate cancer stemness are summarized in this review. In addition, it provides an overview of the models that allow the study of cancer stem cells for the development of new targeted therapies.

**Abstract:**

Tumor heterogeneity represents an important limitation to the development of effective cancer therapies. The presence of cancer stem cells (CSCs) and their differentiation hierarchies contribute to cancer complexity and confer tumors the ability to grow, resist treatment, survive unfavorable conditions, and invade neighboring and distant tissues. A large body of research is currently focusing on understanding the properties of CSCs, including their cellular and molecular origin, as well as their biological behavior in different tumor types. In turn, this knowledge informs strategies for targeting these tumor initiating cells and related cancer stemness. Cancer stemness is modulated by the tumor microenvironment, which influences CSC function and survival. Several advanced in vitro models are currently being developed to study cancer stemness in order to advance new knowledge of the key molecular pathways involved in CSC self-renewal and dormancy, as well as to mimic the complexity of patients’ tumors in pre-clinical drug testing. In this review, we discuss CSCs and the modulation of cancer stemness by the tumor microenvironment, stemness factors and signaling pathways. In addition, we introduce current models that allow the study of CSCs for the development of new targeted therapies.

## 1. Introduction

**Figure 1:**
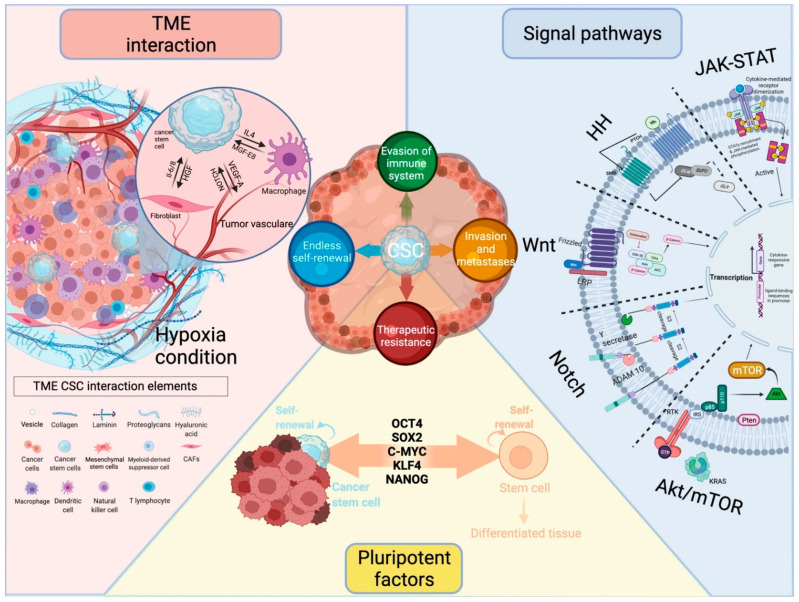
CSCs and the modulation of cancer stemness by the tumor microenvironment, stemness factors, and signaling pathways. Impact of CSCs on tumor progression, recurrence, and resistance to therapy. Created with BioRender.com (accessed on 22 December 2021).

Cancer stem cells (CSCs) are a population of cells present in malignant tumors that share many similarities with normal stem cells or progenitor cells. The common characteristics of these cells include the ability to self-renew and differentiate into multiple lineages, leading to activation of tumor growth and heterogeneity. Mutations that occur in the stem cell pool may contribute to the process of oncogenesis [1]. In addition, there is a link between aging and an increase in the incidence of cancer. There is an accumulation of damage that can lead to the inactivation of tumor suppressor genes and the activation of oncogenes with aging [2]. The origin of CSCs remains unclear, as well as whether they originate from normal stem cells or non-stem cells through re-acquisition of stem cell traits (stemness) through a change in differentiation [3,4,5] (Figure 1). CSCs are generally a rare population of cells, usually amounting to 0.01–2% of the total tumor mass. A notable exception to this evidence is represented by malignant melanoma which comprises a high proportion of CSCs [6]. CSCs share many characteristics with their normal counterparts, including expression of surface markers and regulation by common signaling pathways [7,8,9]. Most CSC surface markers (Table 1) are expressed by embryonic or adult stem cells and are rarely expressed by normal differentiated tissue cells [10]. The expression of surface markers is used to identify and isolate CSCs by fluorescence-activated cell sorting (FACS) and magnetic-activated cell sorting (MACS) [11,12]. Additionally, for the isolation and enrichment of CSCs, the following methods are used: intracellular enzyme activity, promoter-driven fluorescent protein expression, suspension/adherent culture, immunoselection, etc. [13]. However, CSC markers can be characterized not only by the expression of surface markers, but also by intrinsic and extrinsic factors which include transcription factors, microRNAs (for example, miR-21, miR-210, miR-34a, and miR-16) [14], signaling molecules [15,16,17], and extracellular matrix (ECM) [18].

All of these factors regulate CSC function, under the influence of the tumor microenvironment (TME). Differently from normal stem cells, whose proliferation is finely regulated by interactions with their physiological niche, CSCs demonstrate abnormal regulation of self-renewal that enables their expansion either by symmetrical or asymmetrical cell division [7]. Differences between the niche surrounding normal cells and CSCs are at the basis of CSCs ability to transition into different cell states, to remain dormant for long periods of time, and to metastasize to different sites [39]. The niche is a specialized microenvironment that regulates normal stem cell function through cell-to-cell interaction and paracrine signaling. Escape of normal stem cells from control of proliferation and apoptosis provided by the niche is associated with malignant transformation and the formation of a “CSC niche” which induces the recruitment of cells of the TME (mesenchymal stem cells (MSC), immune cells, endothelial cells, and tumor associated fibroblasts (TAF)), secreting growth factors and cytokines essential for sustaining CSC self-renewal [40,41]. By maintaining differentiation capacity, CSC can then lead to the formation of heterogeneous tumors characterized by the growth of phenotypically different subclones [42] of transit-amplifying cells which accumulate and accelerate tumor growth [3]. These heterogeneous cell populations demonstrate high plasticity potential [43,44] and high resistance to stressful factors present in the TME, such as low oxygen or lack of nutrients [39,45].

The CSC niche therefore supports the basic properties of CSCs, preserves their phenotypic plasticity, protects them from the immune system, and contributes to their metastatic potential [39]. Key to the CSC plasticity and metastatic potential is the process of epithelial–mesenchymal transition (EMT) [46,47]. EMT can be induced by the cells of the TME secreting cytokine (e.g., TGFβ and interleukins) to causes changes in cytoskeleton organization, loss of apical-basal polarity, expression of E-cadherin, and acquisition of mesenchymal features and motility, as well as remodeling of the extracellular matrix (ECM) [48,49]. Through these changes, EMT induces a CSC phenotype which enables tumor metastasis and confers resistance to therapy [50,51,52]. It should be emphasized that metastasis is associated with circulating tumor cells that originate from the primary tumor, but these cells have properties of stemness and EMT, which contribute to their penetration and circulation in the blood and increased metastatic ability [53].

Indeed, CSCs are inherently resistant to radiation and cytotoxic drugs, and therefore responsible for minimal residual disease and cancer recurrence [54]. Currently, the study of cancer stemness poses a number of questions, which include: how different are CSCs from normal stem cells; are CSC properties changing during the course of disease; are there differences in stemness between different cancer subtypes; how CSC knowledge can be used to advance precision medicine [55].

In this review, we discuss cancer stemness, CSCs, and their regulation by extrinsic and intrinsic factors. Stemness can be affected by both physical and chemical factors. The main attention of this article is focused on chemical factors, molecular pathways of CSC interaction, and their microenvironment. A deeper understanding of the interactions between CSCs and the microenvironment, including the mechanisms responsible for switching cancer cells from non-CSC to CSC status, is essential for the discovery of effective new therapies. In addition, knowledge about extrinsic and intrinsic factors and their influence on stem formation can provide targeted therapy as well as prevent cancer recurrence and metastasis.

## 2. Pluripotency Factors as Intrinsic Factors Regulating Cancer Stemness

CSC function is determined by a dysregulation of stemness-related signaling pathways. A reduced level of tumor differentiation and increased self-renewal are a characteristic of stemness. Transcription factors which are master regulators of self-renewal and pluripotency in embryonic stem cells (ESCs) have been demonstrated to play a key role in the regulation of stemness in cancer [56]. These transcription factors include the octamer-binding transcription factor 4 (OCT4), the sex-determining region Y-box 2 (SOX2), the homeobox transcription factor NANOG, the Kruppel-like factor 4 (KLF4), and the proto-oncogene C-MYC [57,58,59].

Expression of these factors can reprogram somatic cells into induced cancer stem cells and promote cell plasticity allowing cancer cells to adapt, survive, grow, and resist therapies. This effect has been demonstrated by a recent study showing acquisition of stemness after induced expression of OCT4, SOX2, and NANOG and high expression of pluripotency genes in advanced prostate, bladder, and renal cancers which was correlated with aggressive disease and drug resistance [60]. In addition, pluripotency factors have been shown to mediate cell plasticity in the TME and of enhance ECM production leading to metastasis [61]. Expression of pluripotency factors also regulate the expression of EMT mediators SNAI1 and SNAI2 [62].

Ectopic expression of OCT4 induced a block of differentiation and dysplasia in epithelial tissues [63,64]. Expression of OCT4 has been found in several cancer types and it contributes to the self-renewal and chemoresistance of CSCs [65,66]. Indeed, OCT4 induces the expression of the drug transporter ABCG2, which is highly expressed in CSCs and responsible for drug resistance [67]. Moreover, a relationship between OCT4 translation and metastasis of colorectal cancer to the liver have been demonstrated [68]. Similarly, it has been shown that OCT4 expression in lung cancer cells promotes the polarization of M2 type macrophages due the macrophage colony-stimulating factor (M-CSF) secretion, which leads to increase in tumor growth and metastasis [69].

SOX2 expression is also associated with cancer stemness [70,71]. Expression of this transcription factor is increased in cells and tumor tissue of patients with triple-negative breast cancer (TNBC). Importantly, inhibition of SOX2 suppresses proliferation and invasion of breast cancer cells, inducing cell apoptosis in vitro and inhibiting tumor growth and metastasis in vivo [72]. SOX2 knockout in a mouse model of osteosarcoma also induces a sharp decrease in frequency and occurrence of tumors [73]. In addition, SOX2 and CD133 co-expression can be associated with poor outcome in colon, stomach, and ovarian cancers, as well as melanoma and advanced cancers with bone metastases [74].

NANOG is also involved in maintaining embryonic stem cell self-renewal and cancer stemness [75,76,77]. It has been shown that an increase in the number of oral cancer stem-like cells is associated with increase expression of NANOG and increase malignancy [78]. The expression of this transcription factor increases with the degree of dysplasia and is an early predictor of cancer risk in patients with oral cavity malignant diseases [79]. Mutation in the tumor suppressor SPOP and negative regulator of NANOG also leads to increased stemness of prostate cancer and a negative prognosis in prostate cancer [80]. Dehghan Harati et al. have shown that the expression of NANOG is associated with the increased activity of ALDH and radioresistance, as well as with repair of double-strand DNA breaks [81].

Together with other pluripotency genes, KLF4 plays an important role in the regulation of cell growth, proliferation, and differentiation [82]. In embryonic stem cells, KLF4 activates the expression of telomerase reverse transcriptase (*TERT*) and contributes to the maintenance of self-renewal [83]. In cancer, KLF4 can act either as oncogene by inhibiting apoptosis or tumor suppressor by inducing p21-dependent cell cycle arrest. For instance, KLF4 is highly expressed in a subset of human melanomas and ectopic KLF4 expression enhances melanoma cell growth by decreasing apoptosis [84]. It has also been shown that KLF4 expression is associated with stemness of osteosarcoma [85]. However, KLF4 can also function as tumor suppressor and its knockdown can promote migration and invasion of non-small-cell lung carcinoma (NSCLC) [86].

Similarly, enhanced expression of KLF4 by lentiviral transduction increased sensitivity of ovarian cancer cells to the chemotherapeutic drugs paclitaxel and cisplatin [87].

Finally, C-*MYC* coordinates various biological processes in stem cells, such as cell cycle, cell metabolism, self-renewal, differentiation, and apoptosis [88]. Mutations in *MYC* genes have been found in many tumors and C-MYC is upregulated and acts as an oncogene in more than 50% of human cancers [89]. The expression of C-MYC correlates with the level of differentiation in cancer, as expression of C-MYC induces de-differentiation and acquisition of CSC properties, including glutamine metabolic addiction, dormancy and therapeutic resistance [90]. Dysregulation of MYC usually plays an important role in maintaining the number of invasive CSCs. For example, increased expression of *MYC* is associated with glioblastoma CSC-induced cell proliferation and invasion, and apoptosis inhibition [91].

## 3. Signaling Pathways Modulate Cancer Stemness

Several signaling pathways that are known mediators of juxtacrine (cell–cell) and paracrine extracellular signaling in the local TME have been identified to be key extrinsic players in the regulation of cancer stemness. These include Wnt, Notch, Hedgehog (Hh), Janus kinase/signal transducers and activators of transcription (JAK/STAT), and phosphatidylinositol 3-kinase/serine/threonine-protein kinase/mammalian target of the rapamycin (PI3K/AKT/mTOR) (reviewed in detail in Yang et al.) [56]. Moreover, some of these pathways also participate in epithelial-to-mesenchymal and mesenchymal-to-epithelial (MET) transitions, thus regulating cell identity and plasticity [92].

In addition, there are a large number of studies related to other signaling pathways involved in cancer progression, self-renewal, and metastasis of CSCs [56,93]. For example, recent developments to target and inhibit NF-κB in the ovarian cancer or disruption of the NF-κB/IL-8 signaling in breast cancer can potential targeted therapy for CSCs [94,95,96]. Signaling regulation can be complex in different types of tumors, with cross-interaction of pathways participating in the regulation of CSCs [54,97].

### 3.1. Wnt Signaling

The activation of the Wnt pathway is common in cancer and can be caused by mutations in Wnt signaling components [98,99,100], as well as in downstream targets. Indeed, aberrant activation of Wnt mediators such as APC, β-catenin, Axin, Wnt1, and others are found in many cancers. For instance, thyroid receptor-interacting protein 6 (TRIP6) is an adapter protein that belongs to Lim proteins Zixin family and plays an important role in regulating the function of CSCs in breast cancer through regulation of Wnt/β-Catenin signaling [101]. Similarly, B-cell lymphoma/leukemia 11A (BCL11A) contributes to formation and invasion of tumor cells, stem cell self-renewal and activation of signalling by Wnt/β-Catenin and the EMT pathway. In addition, BCL11A is associated with lung metastasis and increase stemness of breast cancer cells [102].

Interestingly, glioblastoma cells expressing high levels of Wnt demonstrated expression of OCT-4, SOX2, NANOG, NESTIN, and CD133, thus suggesting a role of Wnt signaling in the maintenance of glioma CSCs [103].

### 3.2. Notch Signaling

The Notch pathway is also important for CSC function, and it is activated in tumors surviving and adapting to their microenvironment. Activation of the Notch pathway contributes to self-renewal, metastasis, and suppression of apoptosis. For example, the aberrant transmission of Notch signals (Notch1 and Notch4) contributes to self-renewal and metastasis of breast CSCs [104]. High levels of Notch1, Notch3, JAG1, JAG2, and the target HES-1 are found in pancreatic and breast cancers [105,106]. Notch signaling is activated under hypoxic conditions in breast cancer mediating chemoresistance and CSC expansion, which can be reversed by treatment with Notch inhibitors [107]. In addition, suppression of Notch1 via miR-34a can lead to an increase in breast cancer cell chemosensitivity to paclitaxel with a reduction in CSC proliferation and expression of the stemness marker ALDH1 [108]. Glioma stem cells are also regulated by activation of Notch1 and they show increased expression of the CSC genes OCT4 and CD133 under hypoxia [109].

### 3.3. Hedgehog Signaling

Together with Wnt and Notch signaling, the Hedgehog pathway is involved in embryonic development and organogenesis, including the nervous system, and organs such as lung, heart, and bowel [110]. Abnormal activation of the Hedgehog signaling pathway can be detected in CSCs [111,112]. For instance, it contributes to self-renewal, proliferation, and tumorigenicity of lung adenocarcinoma stem cells [113]. Through activation of the PTCH1 receptor and downstream effector Gli-1, Hedgehog signaling stimulates the transcription of the target genes OCT4, SOX2, NANOG, and C-MYC [114]. Zhu et al. showed that SHH, PTCH1, and Gli-1 are activated by TSPAN8 expression in breast CSCs leading to increased expression of NANOG, OCT4, and ALDHA1 genes, as well as increased stem cell self-renewal and cell survival after treatment with adriamycin and paclitaxel [115]. Similarly, Hedgehog signaling stimulates self-renewal of glioma CSCs as they overexpress SHH, PTCH11, and GLI1 [116]. The Hedgehog pathway has also been shown to be important for pancreatic CSCs, as inhibition of the ligand SHH by inhibition of sialidase-2 (Neu2) and desialylation leads to a decrease in stemness [117].

### 3.4. JAK/STAT Signaling

The JAK/STAT pathway promotes survival, self-renewal, hematopoiesis, and neurogenesis of ESCs [118]. This pathway is also activated in CSCs [119]. Among the different subtypes of STAT proteins, activation of STAT3 plays an important role in CSC function by regulating oncogenic signaling pathways. STAT3 is constitutively activated in many different cancers, including pancreatic breast, prostate, ovarian liver, colorectal, and bone cancers, as well as leukemia and melanoma. In addition, STAT3 activation is associated with the generation of glioblastoma stem cells and the metastatic potential of colon CSCs [90]. As well as STAT3, it has been shown that suppression of STAT1 reduces the formation of lung A549 tumor spheres which was maintained by the suppression of factors associated with stemness, such as SOX2, OCT4, and NANOG [120].

### 3.5. AKT/mTOR Signaling

The PI3K/AKT/mTOR signaling pathway is important for cell proliferation and survival, and abnormal activation of PI3K/mTOR signals is commonly found in cancer [121,122]. The activation of this pathway also increases the migration, invasion, and resistance of the CSCs [123]. The transmission of PI3K/AKT signals is part of the main molecular stemness program both in mouse and human pluripotent stem cells. The oncogenic version of PIK3CA^H1047R^ in cancer causes constitutive activation of the PI3K pathway and is associated with increased stemness in a dose-dependent manner, as shown in mouse models of breast, lung, and colorectal cancers [124]. Activation of the PI3KCA is also associated with induction of EMT and stem cell plasticity through multiple signals, including TGFβ [49].

## 4. Influence of the Microenvironment on CSC

Stem cells cannot survive outside their niche environment or in the absence of specific pluripotency factors and signaling pathways that support stem cell function [125]. Importantly, these factors can facilitate the emergence of stem cells from more differentiated cells, as these retain the ability to dedifferentiate and return to a more primitive developmental state [126].

The plasticity demonstrated by cancer cells is key in cancer as extrinsic factors can promote the acquisition of stemness by reprogramming cancer cells into CSCs. These factors include cytokine and growth factors secreted cells of the TME (mesenchymal stem cells (MSCs), macrophages, tumor-associated fibroblasts (TAFs)), as well as extracellular vesicles (EVs), and hypoxia [127]. In epithelial tissues, the activation of EMT has been linked to the formation of both normal cells and CSCs [128]. Fundamental to the process of gastrulation during embryo development, EMT is activated in the adult during wound healing and in cancer [129].

EMT is a reversible process with cells changing phenotypes from epithelial to mesenchymal and then back to epithelial through MET. These highly dynamic processes are regulated by paracrine signaling, most notably TGF-β, Wnt, and others involved in maintaining stem cell function, as described above. These pathways then induce expression of factors triggering EMT, including transcription factors of the TWIST, SNAIL, and ZEB families, splicing factors and microRNAs (e.g., miR34, miR200) which drive the loss of expression of adhesion molecules such as E-cadherin (encoded by the *CDH1* gene), as well as the acquisition of mesenchymal markers, such as Vimentin [130].

Phenotypic plasticity linked to EMT has important implications for CSCs and their cellular origin in different tumor types. For instance, both epithelial and mesenchymal cells in the human breast can adopt a CSC phenotype and co-exist in tumor. Indeed, epithelial CSCs are proliferative and express ALDH, whereas mesenchymal CSCs are mostly quiescent and display a CD44hi/CD24- profile [131]. This dynamic equilibrium is regulated by the TME and the resulting heterogeneity is at the basis of the existence of different disease molecular and pathological subtypes in most solid tumors [42].

Factors associated with inflammation, such as tumor necrosis factor (TNF), interleukin-6 (IL-6), and IL-1β, can activate EMT [132]. For instance, IL-6 serum levels are high in osteosarcoma patients and the cytokine stimulates osteosarcoma stemness as measured in a self-renewal spheroid assay [133]. It was also found that IL-1β can increase the formation of colon cancer spheres, which show an up-regulation of stemness factor genes and increased drug resistance [134]. Finally, tumor necrosis factor (TNF)-α promotes HPV-associated oral carcinogenesis by increasing stemness [135].

These signaling pathways are also involved in the communication between cancer-associated fibroblasts (CAFs) present in the tissue stroma and cancer cells. Indeed, CAFs can activate signaling promoting cancer stemness through activation of Wnt and Notch signaling. CSCs, in turn, can influence CAFs through activation of signals involved in cancer progression, including the Hedgehog pathway [136]. Inter-related signaling pathways also link hypoxia with EMT. Indeed, hypoxia can directly induce EMT via the activation of the hypoxia-inducible factor (HIF)-1α through cross-talk with TGFβ and Wnt/β catenin pathways. In addition, hypoxia can also induce EMT via HIF-independent pathways which include AMPK, PIK/AKT, MAPK, NF-kB, and Notch signalling [137].

Other non-cellular components of the TME can modulate CSCs, including ECM and EVs. Among ECM molecules, tenascin-C is involved in the stimulation of self-renewal of CSCs. In breast cancer, it promotes stemness through upregulation of the CSC marker LRG5 [138] and it is also associated with poor prognosis in glioblastoma and represents a candidate CSC markers in this cancer type [139]. In addition, the ECM provides a physical barrier to CSCs from cytotoxic drugs and may promote EMT, self-renewal, expression of CSC markers, and drug resistance. ECM properties such as stiffness and porosity affect various CSC functions. The rigidity of the ECM is involved in the regulation of self-renewal and differentiation of stem cells [140,141]. Tumor ECM is usually more rigid than normal tissue ECM due to overexpression of collagens, proteoglycans, and ECM-modifying enzymes (lysyl oxidases) [142].

Finally, EVs isolated from tumor and stromal cells are involved in various stages of tumor progression such as proliferation, angiogenesis, metastasis, and drug resistance [143]. Tumor cells secrete a heterogeneous set of EVs, which differ in size, biogenesis, and molecular composition, which include cytoplasmic proteins, proteins interacting with lipid rafts, DNA, and RNA [144]. Communication through EVs is important for the maintenance of CSCs. For instance, Evs released by glioblastoma stem cells promote self-renewal and angiogenesis through endothelial tube formation [145]. Similarly, exosomes derived from TAFs promote the formation of colorectal cancer spheres by activating Wnt signaling and ultimately increasing the number of CSCs [146]. Gonzalez et al. also showed that stem/progenitor-enriched mammospheres from primary mammary epithelial cells can secrete extracellular vesicles that are capable of altering the expression levels of genes involved in EMT and stem cell markers [147].

## 5. Models of Cancer Stemness and TME Interaction

Culture models that allow cancer cells to re-acquire or enrich for stem cell characteristics have been developed to study CSCs. These culture models are aimed at preserving the biological characteristics of primary tumors, thus exhibiting the original tumor architecture and metabolic activity [148]. They also provide more accurate data on the effects of therapeutic agents and processes such as EMT and MET [149].

Among the 3D culture model systems which allow CSC self-renewal are spheroids and organoids cultured in suspension, scaffolds, matrix, and hydrogel cultures.

### 5.1. Spheroids

Spheroids are 3D tumor models that can be obtained by aggregation or spontaneous self-assembly of cancer cells. Aggregation can be caused by forced cell-to-cell contact by various methods such as hanging drop, liquid application/cell suspension culture, microwell culture (96-well round bottom plates), or microfluidics (i.e., gel encapsulation) [150]. Tumorsphere cultures are mainly generated by single cancer cell suspension in serum-free media. Under these conditions, only cells with self-renewal characteristics allow the formation of cell aggregates or spheroids that can proliferate and be serially passaged [151,152]. Spheroid cultures have been established from many different cancer types, including breast, prostate, bone, skin, brain, and colon tumors. Biomaterials can be used to support 3D CSC models, as they provide a physical structure to create a niche environment which is important to better sustain CSC function. Porous scaffolds are most commonly made of synthetic (polyglycolic and polylactic acids) or natural (collagen, alginate) polymers and hyaluronic acid. These have been used to support CSC proliferation in prostate and breast cancer, hepatocellular carcinoma, and glioblastoma. Hydrogels (natural and synthetic) are also widely used for 3D models of CSCs and they can be coupled with the use of extracellular matrix [153].

### 5.2. Organoids

Differently from spheroids, organoids are 3D cultures of stem cells that enable the differentiation and self-organization of tissue-like structures in vitro. These cultures are supported by embedding of cells in matrix and growth factors that normally support the survival and growth or stem cells in their natural niche. Organoids can be derived from cells isolated from primary tumors and many studies have demonstrated their ability to maintain tumor heterogeneity and pathological characteristics, even after multiple passaging. Organoids have been already established from a range of tumors, including colon, breast, pancreatic, liver, and prostate cancer. For example, 3D epithelial organoids can be established clonally from LRG5-positive intestinal stem cells and colon cancer cells [154].

Another application of organoids in CSC research is the use of iPSC technology and gene editing. Indeed, organoids can be generated through differentiation of gene-edited normal iPSC to carrying cancer driver mutations as well as iPSC derived from tumor cells isolated from cancer patients. The cancer iPSC-derived organoids have been shown to contain CSC that establish heterogeneous tumoroid structures that recapitulate primary tumors [155].

### 5.3. TME Interaction

Although the described models are powerful tools to maintain and enrich CSCs ex vivo, they face the challenge of mimicking the intra-tumor heterogeneity of primary tumors due to the modulation of CSC by their microenvironment. The bidirectional crosstalk between CSC and TME maintains cancer stemness. Niche cells of the TME communicate with CSC inducing self-renewal and tumor progression. Physical characteristics of the CSC niche, such as hypoxia and interaction with ECM, also affect CSC function [156]. These features can be modeled in vitro to some extent and current research efforts are directed to create model systems that closely resemble patient’s cancer complexity. Histomorphological analysis shows that the spheroid model is divided into several zones, like a primary tumor. The outer marginal zone is characterized by preferentially proliferating cells, the middle zone by dormant cells, and the central part by a necrotic core due to lack of oxygen and nutrients. The central part of spheroids is characterized by the accumulation of lactate due to the significant consumption of glucose in the proliferating layer of cells whilst the distribution of glucose occurs evenly in all areas of spheroids [157]. Glycolytic metabolism and hypoxia are critical for maintaining stemness of CSCs. A hypoxic microenvironment can also activate CSC signalling. For instance, breast CSCs in mammospheres activate Wnt and Notch signalling in hypoxia [107]. This is mirrored by glioblastoma and colorectal CSC activating Notch, Hedgehog, and Wnt pathways [158]. ECM proteins play an important role in the modulation of stemness in CSC models. In a 3D model of colon cancer using afibrin gel, CSC properties were enriched with the activation of NANOG [133]. Similarly, collagen type I (Col-I) oligomer 3D matrices induce stemness and EMT in pancreatic cells [159].

Interactions with cells of the TME can be effectively modelled to study modulation of CSCs. Breast cancer cells can adopt a proliferative and EMT phenotype when cultured in 3D spheroids in the presence of mesenchymal stem cells (MSCs) [160]. The effect of MSCs on tumor cells has also been studied by creating hybrids with gastric cancer cells using polyethylene glycol (PEG) in vitro. These hybrids show a change in EMT with down-regulation of E-cadherin and up-regulation of vimentin, N-cadherin, α-smooth muscle actin (α-SMA), and fibroblast activation protein (FAP). Hybrids increase the expression of the stemness factors OCT4, NANOG, SOX2, and LIN28, upregulate CD44 and CD133 as well as promote gastric xenograft tumor growth in vivo [161].

Therefore, the co-culture of cancer cell in 3D with cells of the TME can model the complexity of patient’s tumors which, combined with the use of microfluidic devises and 3D bioprinting, can be used to investigate many aspects of tumor stemness.

As the eradication of CSCs is clinically important, the study of CSCs in a TME context can provide invaluable clues in the fight against cancer, such as the discovery of new biomarkers for early detection of cancer and effective therapies [162,163].

## 6. Conclusions and Future Perspectives

The survival rate of cancer patients has increased significantly over the past decades, thanks to the development of anticancer therapy which includes interdisciplinary care, improved chemotherapy drugs, and targeted biological agents. However, despite these advances, the problems of cancer relapse and treatment of advanced-stage tumors remains unresolved. Currently, there is no therapy that would effectively treat patients with advanced metastatic disease. The main factors that complicate the treatment of patients are cellular heterogeneity, as well as the structural and molecular complexity of the tumors. Intratumor heterogeneity arises from genetic mutations, interactions with the microenvironment, and the presence of tumor stem cells. Activation of signaling pathways that promote self-renewal and chemoresistance allow CSCs not only to successfully survive unfavorable conditions but also to self-renew with the production of resistant clones. Therefore, targeting cancer stemness is an increasingly important therapeutic approach to halt tumor progression, recurrence, and resistance to therapy.

To this end, there is some evidence that some compounds have a direct effect on CSCs. For instance, salinomycin, a potassium ionophore, can selectively kill breast cancer stem cells and induce epithelial differentiation of breast tumor cells, promoting effective tumor removal [164]. In addition, epigallocatechin gallate reduces the stem and oncogenicity of human lung cancer cells by inhibiting AXL receptors [165].

Although promising, data of effective CSC therapies are limited and therefore the development of CSC-specific therapies requires better knowledge of the relationship between CSCs and their microenvironment. A deeper understanding of the intrinsic and extrinsic factors involved in shaping this interaction is likely to be essential to overcome the barrier of CSC plasticity. Although therapeutic strategies aimed at inhibiting the signaling pathways of CSC are available (Table 2), it is necessary to continue researching new strategies that can target factors inducing cancer stemness, including TME (EMT, hypoxia, CAFs, vesicles), metabolism, and chemoresistance [166].

However, a significant obstacle to the development of effective therapies that can take into account cancer complexity is the discrepancy between model systems and patient’s tumors. Modelling of the TME is especially complex and therefore the use of clinically relevant and close to patient models can address the probe of the high attrition rate in cancer drug development. In addition, the combination of different models can add to their predictive value. For instance, the use of CSCs to create cultures of non-adherent spheres, three-dimensional tumor organoids, and patient-derived xenografts have shown that treatment with Wnt and Notch inhibitors can block the proliferation and self-renewal of CSCs [11].

Thus, the use of advanced and complex model systems will expand the possibilities of CSC therapy, which will stimulate the development of precision medicine.

## Figures and Tables

**Table 1:** CSCs markers in different types of tumors.

Cancer Type	CSCs Markers	Reference
Blood tumors	CD34^+^ CD38^−^ phenotype	[19]
Brain tumors	CD133^+^, CD49f^+^, CD90^+^, epidermal growth factor receptor (EGFR)^+^, c-series ganglioside-specific antigen A2B5^+^, L1 cell adhesion molecule (L1CAM)^+^	[20,21,22]
Ovary tumors	CD24^+^, aldehyde dehydrogenase (ALDH)^+^, CD44^+^/CD117^+^, epithelial cell adhesion molecule (EpCAM)^+^, CD133^+^	[23,24]
Prostate tumors	EpCAM^+^, CD117^+^, α2β1 integrin^+^, ALDH^+^, CD44^+^, enhancer of zeste homolog (EZH)^+^, CXC chemokine receptor type 4 (CXCR4)^+^, E-cadherin^+^, CD133^+^	[13,25,26]
Colon tumors	CD133^+^, CD44^+^, CD166^+^, CD24^+^, EpCAM^+^),	[27,28,29,30]
Pancreatic tumors	CD133^+^, CD44^+^, CD24^+^, EpCAM^+^, tyrosine-protein kinase Met (cMet)^+^	[31,32]
Liver tumors	CD44^+^, CD90^+^, CD206^+^, oval cell antigen 6 (OV-6)^+^), skin (CD20^+^, CD271^+^, ALDH^+^, CD133^+^	[33]
Lung tumors	CD133^+^, ATP-binding cassette super-family G member 2 (ABCG2)^high^, CD166^+^, CD90^+^, CD87^+^, ALDH^+^, CD44^+^	[34]
Breast tumors	ALDH1^+^, CD24^+^, CD44^+^, CD90^+^, CD133^+^, α6-integrin^+^	[35,36,37,38]

**Table 2:** Therapeutic agents targeting cancer stem cells.

Agent	Notes	Clinical Trials	Reference
MRK-003, MK-0752, R4733	Notch inhibitors	NCT00106145 NCT01154452	[167,168,169]
CAL-101, XL-147	PI3K inhibitors	NCT01629615 NCT01613950 NCT00907205	[170,171]
KRX-0401, RX-0201	AKT inhibitors	NCT00590954 NCT01028495	[172,173,174]
BMS-863923, IPI-926	Hedgehog inhibitors	NCT01546038 NCT01130142 NCT01700049	[175]
OMP-54F28, PRI-724, CWP232291	Wnt inhibitors	NCT01606579 NCT01351103 NCT02092363	[176,177,178]
BBI503, BBI608	NANOG inhibitors	NCT02232633 NCT02315534	[179,180]
